# First Branchial Cleft Cyst Confined to the Pinna

**DOI:** 10.7759/cureus.36829

**Published:** 2023-03-28

**Authors:** Alison C Ma, Beatrice R Bacon, Iris Danziger, Michele M Carr

**Affiliations:** 1 Otolaryngology, Jacobs School of Medicine and Biomedical Sciences, University at Buffalo, Buffalo, USA

**Keywords:** clinical case report, first branchial cleft, branchial cleft anomaly, first branchial cleft cyst, branchial cleft cyst

## Abstract

First branchial cleft cysts (FBCCs) arise due to an incomplete fusion of the cleft between the first and second branchial arches. Classically, they are found inferior to the pinna or along the external auditory canal. This report presents a unique case of a nine-month-old male with a first branchial cleft cyst completely within the pinna. The patient presented with a left auricular pit and pinna mass. Ultrasound revealed a homogeneous hypoechoic mass isolated to the pinna. Surgical resection revealed the cyst to be anterior to the inferior pinna cartilage, with the tract projecting anteriorly and inferiorly. Final pathology revealed a benign cyst lined by squamous epithelium and a rim of cartilage, confirming an FBCC. To our knowledge, FBCCs isolated to the pinna have not been previously reported. Awareness of the various presentations of this rare anomaly is essential for a prompt and accurate diagnosis.

## Introduction

First branchial cleft cysts (FBCCs) arise due to a developmental defect of the first branchial cleft and arch. They are rare, representing only 1% of all branchial anomalies [1]. They are typically found inferior to the pinna along the external auditory canal or in the region of the angle of the mandible [2]. The first sign is usually a swelling with or without drainage. Diagnosis is largely clinical with confirmation via pathology. However due to their rarity, they can be easily misdiagnosed and mistreated. Imaging via ultrasound (US), computed tomography (CT), fistulography, and magnetic resonance imaging (MRI) can be performed to help localize the cyst and tract. Treatment typically consists of complete resection of the tract to prevent a recurrence. Since the original characterization of FBCCs, numerous cases have been published to increase awareness of the various presentations. Here, we discuss a unique presentation of an FBCC within the pinna.

## Case presentation

**Figure 1:**
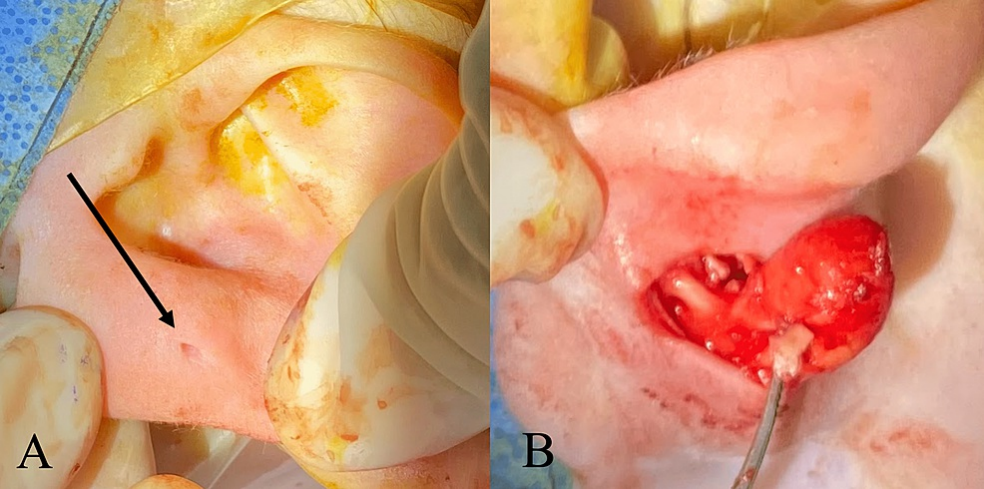
Intraoperative images of the (A) pre-auricular pit (arrow) and (B) dissection of the cyst with the tract projecting anteriorly. The cyst was measured to be 1.5 x 1 x 0.4 cm.

**Figure 2:**
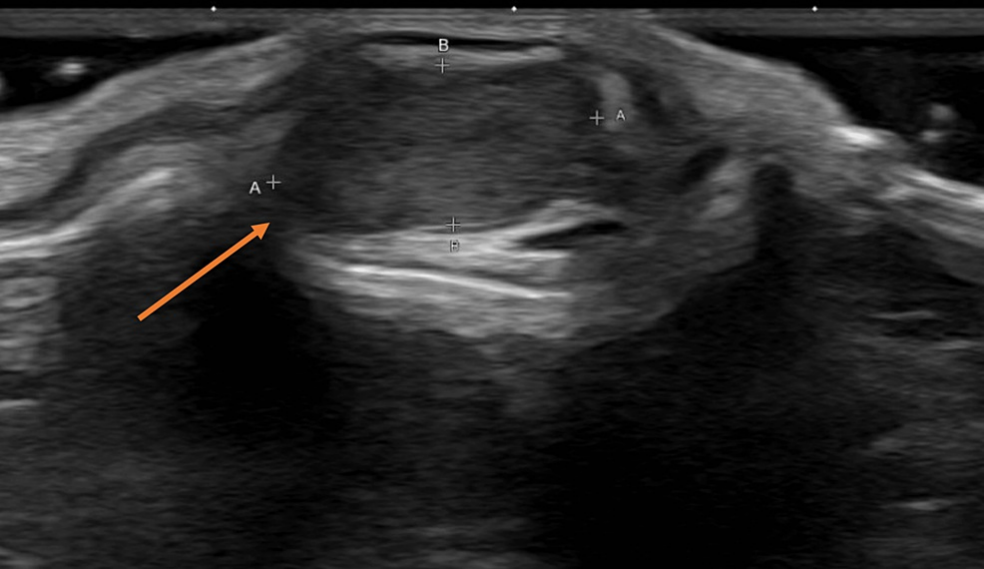
Ultrasound examination of the cyst on the left auricular cyst showing homogeneous, well-circumscribed, hypoechogenic mass (arrow).

A nine-month-old infant presented with a history of a left pre-auricular pit since birth (Figure 1A) and the presence of a left posterior auricular lump since six months of age. His parents denied any change in size, drainage, or notable irritation. The patient had an unremarkable medical history but had a family history of neonatal brain tumors. Physical exam revealed a non-tender 1.5-cm mass on the left pinna with a normal post-auricular region. The left external auditory canal and tympanic membrane were normal on inspection. The right ear exam was unremarkable. US revealed a homogeneous, hypoechogenic, well-circumscribed mass isolated to the left pinna (Figure 2). Clinical suspicion was significant for a benign cyst, likely a dermoid cyst or a lymph node, but due to the family history of brain cancer, immediate resection was performed. Surgical resection revealed a cyst anterior to the patient’s pinna cartilage, with an anteriorly projecting tract (Figure 1B). Final pathology identified a benign cyst lined by squamous epithelium with adnexal structures and a rim of cartilage, which confirmed an FBCC. On follow-up, the patient appeared to be healing well with no recurrence. This report is written and published with the consent of the patient’s family.

## Discussion

FBCCs are rare with an estimated incidence of one in 100,000 live births [3]. They make up only 5% to 25% of all branchial cleft cysts [2]. When they do present, they are usually located in Poncet’s triangle, which is bordered by the external auditory canal, the hyoid body, and the mandibular angle [4,5]. While the exact mechanism of cyst formation is unknown, it has been hypothesized that branchial cleft cysts result from components of the cervical sinus of His becoming entrapped without an opening [3]. Numerous reports have been published on unique presentations of branchial cleft cysts. This is the first report, to our knowledge, of a patient with an FBCC isolated to the pinna. 

Branchial (pharyngeal) structures begin to develop early in the fourth week of gestation to become components of various head, neck, and thorax structures. They start as four clefts that form five mesodermal ridges, which later become six branchial arches. The arches then externally form grooves or clefts and internally form pouches. Each arch ultimately transforms into a component of an anatomic structure. Branchial cleft cysts arise following incomplete fusion of branchial arch structures. The first branchial arch becomes the mandible, a portion of the maxillary process of the upper jaw, and a portion of the inner ear. The first branchial cleft and pouch become the external auditory canal (EAC), eustachian tube, middle ear cavity, and mastoid air cells. FBCCs are commonly found adjacent to the EAC. This contrasts the more common second branchial cleft cysts which comprise up to 95% of BCC cases [6]. These are commonly found along the sternocleidomastoid muscle. The second arch forms the hyoid bone and its surrounding structures, and its pouch becomes the palatine tonsil and supratonsillar fossa. Third-arch anomalies are uncommon and found anterior to the sternocleidomastoid, and fourth-arch anomalies are rare, presenting low in the neck, usually anterior to the sternocleidomastoid. 

The pinna develops along with the EAC from the first two branchial arches. Each arch forms three hillocks, which ultimately form the tragus, helix, concha, and antihelix. It first develops in the lower cervical region and then migrates posterolaterally to its final position. It is along this course that the FBCC becomes intertwined with the facial nerve and parotid gland [7]. Given the unique presentation of this patient’s cyst formation on the pinna, one can speculate whether the so-called error occurred early on when the first branchial cleft and arch were still in close proximity, or later on when they had reapproximated. 

In 1972, Work proposed two types of first branchial cleft cysts: type I and type II lesions [8]. Type I lesions are epidermoid with no cartilage and found superior to the facial nerve [9]. They are due to a duplication of the membranous portion of the external ear. Type II lesions are more common and consist of duplication of both the membranous and cartilaginous portions of the ear and are found in Poncet’s triangle [10]. Work type I cysts appear as duplications of the external auditory canal due to their positioning. They typically present anterior, medial, and parallel to the external auditory canal, lateral to the facial nerve, and end at the middle ear. Work type II cysts generally appear in the submandibular area or at the angle of the mandible. These can course medial or lateral to the facial nerve. 

Clinically, FBCCs typically present with swelling and sometimes purulent drainage through an open tract. Often, a pit or sinus tract can be visualized, as seen in this patient. Although a benign pathology was presumed for this patient, clinical suspicion was low for a branchial cleft cyst given the unusual location. 

Imaging studies can help determine the best surgical approach, though there is no clear imaging technique that is universally recommended. US is generally the first-line modality given its ease of use and avoidance of radiation in young children. CT and fistulography introduce radiation exposure, while MRI requires sedation of young patients, making US the most practical option [11]. CT and MRI have both been praised for their ability to visualize sinus tracts, but generally, children are more tolerant of CT imaging [12,13]. US of an FBCC will reveal a homogeneous hypoechogenic cyst. In this patient, US was used to describe the depth of the cyst and reassured the patient’s parents that the pathology was likely benign. If the lesion had been invasive, heterogenous, and/or poorly circumscribed, with the patient’s family history of malignancy more concern would have been raised for cancer. 

Complete surgical resection is regarded as the definitive treatment, as infection and the need for incision and drainage procedures can lead to scar tissue, fistula formation, and ultimately recurrence of the cyst. Additionally, surgical pathology ultimately still serves the important role to allow us to confirm our diagnosis of a branchial cleft cyst. Resection is ideally performed when the patient is free of infection and inflammation, sometimes requiring an antibiotic course prior to operation. FBCCs can often involve the facial nerve, making preservation of the nerve a vital part of the surgery. In this case, given the isolated location to the pinna, a simple resection was possible without the risk of injuring the facial nerve and parotid gland. Final surgical pathology ultimately still serves as the gold standard in making the diagnosis of branchial cleft cysts.

## Conclusions

We present a unique case of a first branchial cleft cyst completely within the pinna. Although mostly benign and asymptomatic, prompt diagnosis of FBCCs allows for resection of any possible cases that may become symptomatic. This case highlights the importance of considering branchial cleft cysts in the differential diagnosis of pinna masses, as the anatomical location of first branchial cleft cysts may be variable. 
